# Research on prognostic risk assessment model for acute ischemic stroke based on imaging and multidimensional data

**DOI:** 10.3389/fneur.2023.1294723

**Published:** 2023-12-19

**Authors:** Jiabin Liang, Jie Feng, Zhijie Lin, Jinbo Wei, Xun Luo, Qing Mei Wang, Bingjie He, Hanwei Chen, Yufeng Ye

**Affiliations:** ^1^Postgraduate Cultivation Base of Guangzhou University of Chinese Medicine, Panyu Central Hospital, Guangzhou, China; ^2^Graduate School, Guangzhou University of Chinese Medicine, Guangzhou, China; ^3^Medical Imaging Institute of Panyu, Guangzhou, China; ^4^Radiology Department of Sun Yat-sen Memorial Hospital, Sun Yat-sen University, Guangzhou, China; ^5^Laboratory for Intelligent Information Processing, Guangdong University of Technology, Guangzhou, China; ^6^Kerry Rehabilitation Medicine Research Institute, Shenzhen, China; ^7^Stroke Biological Recovery Laboratory, Spaulding Rehabilitation Hospital, Teaching Affiliate of Harvard Medical School, Charlestown, MA, United States; ^8^Panyu Health Management Center, Guangzhou, China

**Keywords:** prognostic risk, assessment model, acute ischemic stroke, imaging, multidimensional data, MRI

## Abstract

Accurately assessing the prognostic outcomes of patients with acute ischemic stroke and adjusting treatment plans in a timely manner for those with poor prognosis is crucial for intervening in modifiable risk factors. However, there is still controversy regarding the correlation between imaging-based predictions of complications in acute ischemic stroke. To address this, we developed a cross-modal attention module for integrating multidimensional data, including clinical information, imaging features, treatment plans, prognosis, and complications, to achieve complementary advantages. The fused features preserve magnetic resonance imaging (MRI) characteristics while supplementing clinical relevant information, providing a more comprehensive and informative basis for clinical diagnosis and treatment. The proposed framework based on multidimensional data for activity of daily living (ADL) scoring in patients with acute ischemic stroke demonstrates higher accuracy compared to other state-of-the-art network models, and ablation experiments confirm the effectiveness of each module in the framework.

## 1 Introduction

Stroke is a cerebrovascular disease characterized by localized cerebral ischemia, hypoxia leading to ischemic necrosis or softening, and subsequent neurological dysfunction. Approximately 16 million people worldwide suffer from stroke each year, with 5.7 million deaths and around 5 million disabilities (1). Survivors often experience difficulties in swallowing, speech impairment, motor dysfunction, cognitive impairment, emotional disorders, and other functional deficits (2, 3). Early diagnosis, prediction, and rehabilitation are key strategies for improving the prognosis of stroke patients. Stroke treatment guidelines emphasize that early diagnosis of stroke relies on imaging findings and clinical symptoms/signs. Neuroimaging plays a crucial role in the definitive diagnosis of suspected stroke patients. However, limited studies have been conducted on the correlation between imaging-based predictions of complications in acute ischemic stroke, and most of them focus on single complications (4, 5). Several scales have been used clinically to predict functional outcomes in stroke patients (6, 7), such as the acute stroke registry and analysis of Lausanne (ASTRAL) and the totaled health risks in vascular events (THRIVE). However, these scales mostly incorporate variables at admission and are intended to provide information for treatment, without collecting post-treatment data for prediction. Therefore, in order to accurately assess the prognostic outcomes of patients, adjust treatment plans in a timely manner for those with poor prognosis, and intervene in modifiable risk factors, machine learning methods are needed to predict the prognostic risk of patients with acute ischemic stroke.

MRI is one of the crucial tools for evaluating acute ischemic stroke and has been widely used in clinical practice due to its high detection accuracy, sensitivity, and specificity. Computer-aided diagnosis (CAD) based on MRI has received extensive attention from researchers both domestically and internationally. For example, the texture analysis of apparent diffusion coefficient maps and diffusion-weighted imaging were used to predict the prognosis and subtype of ischemic stroke (8–10). A systematic review also demonstrated that a combined model combining clinical and imaging variables was more predictive of stroke outcome (11).

**Figure 1:**
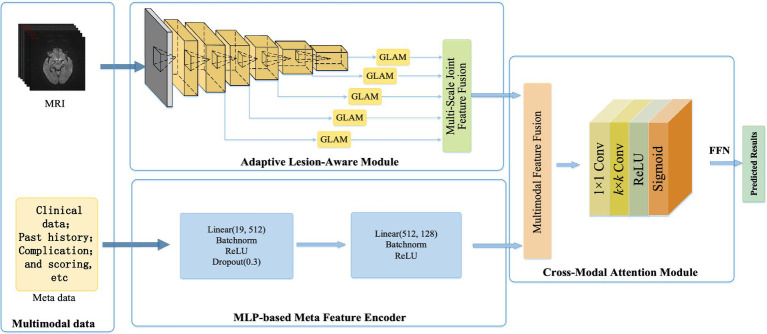
Based on the multi-dimensional network framework for the ADL score in patients with acute cerebral infarction.

This study aimed to construct an acute ischemic stroke prognosis assessment model based on multidimensional imaging data, clinical information, treatment plans, prognosis, and complications, as shown in Figure 1. It contains the adaptive lesion awareness module (ALAM), the patient metadata encoder based on multilayer perceptron (MLP-FE), the cross-modal attention module (CMAM). This model provided a scientific basis for early clinical intervention, enabling healthcare professionals to make informed decisions and interventions based on the predicted prognosis.

## 2 Multidimensional ADL scoring network framework for acute ischemic stroke patients

**Figure 2:**
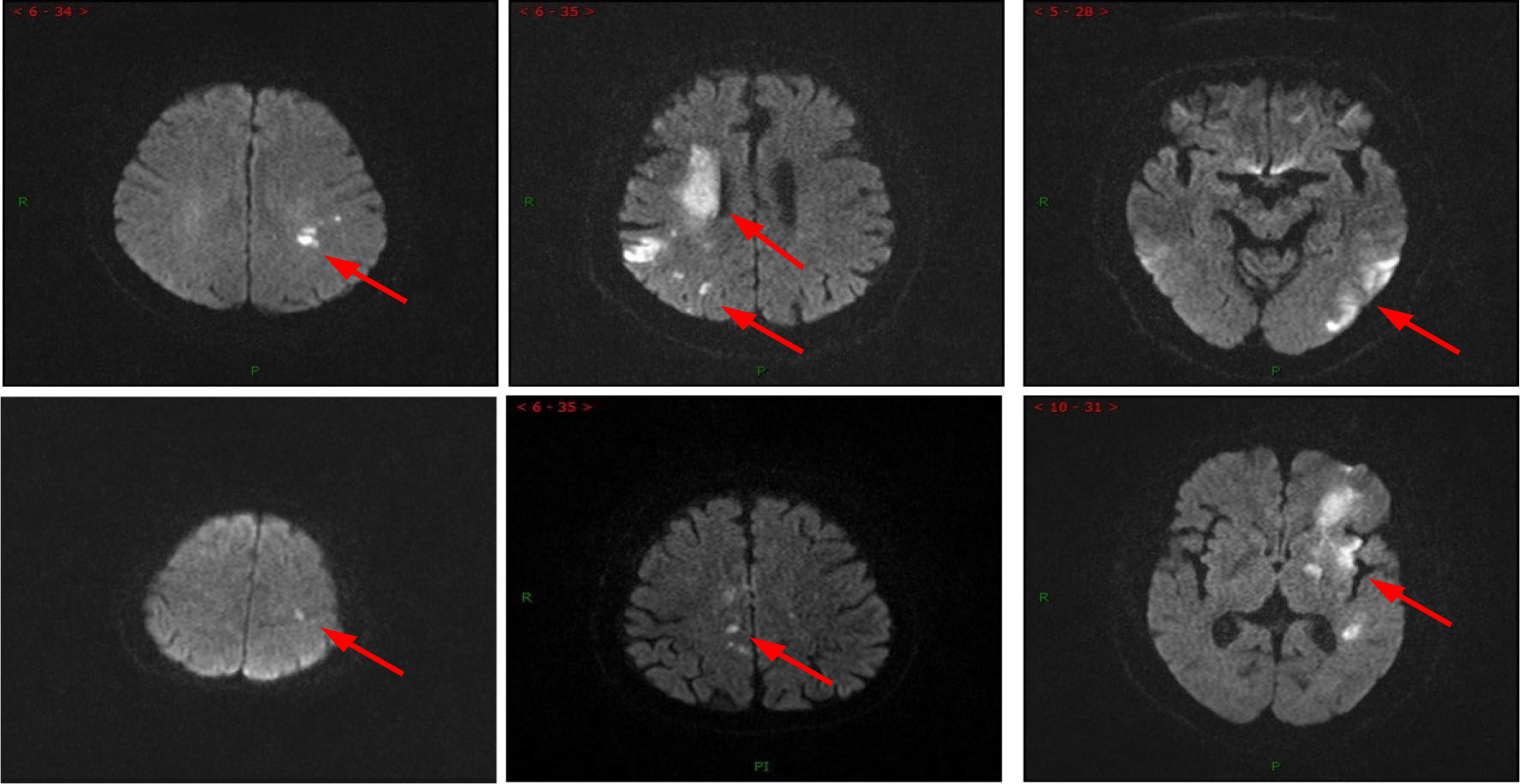
Example of the AIS patient MRI, where the red arrow indicates the focal area.

In this section, we developed a multidimensional data-based cross-modal attention fusion network for the prognosis assessment of acute ischemic stroke patients. The structure of the network was illustrated in Figure 2 and consisted of three main components: the ALAM, the MLP-FE, and the CMAM, which were described as follows:

Firstly, the ALAM was based on a multi-scale global-local attention mechanism. It adaptively focused on the lesion region by learning joint features of global context information and local detailed features, enabling the extraction of more discriminative imaging features (shown as Figure 1).

Secondly, to fully utilize the patient metadata, we designed the MLP-FE. This encoder compiled the patient metadata into corresponding feature representations.

Lastly, the CMAM was proposed in this study for the fusion of multidimensional data, including clinical information, imaging features, treatment plans, prognosis, and complications. It aimed to achieve information complementarity and cross-validation by integrating data from different modalities.

### 2.1 Adaptive lesion sense module

In clinical practice, acute ischemic stroke patients exhibited complex MRI imaging features (shown in Figure 2): the location, size, and appearance of the lesions varied significantly among different patients and stages of development. Furthermore, the complications associated with stroke often had similar imaging characteristics, making them difficult to differentiate. Additionally, mild cases with smaller lesions were prone to being overlooked. This posed a significant challenge for deep learning models to learn lesion imaging features from MRI, as it involved the issues of “same disease, different images” and “different diseases, same images.”

In this subsection, we constructed an integration of the global-local attention module (GLAM) and the multiscale joint feature fusion module. This integration allowed for adaptive attention towards the relevant lesion regions and extraction of MRI images features by combining global and local views, akin to the process of radiologists’ image interpretation. Additionally, the hierarchical design within this module facilitated multiscale joint feature fusion, enabling the extraction of lesion characteristics of different shapes and sizes. Moreover, these features interacted with each other to obtain the most effective image information.

#### 2.1.1 The global-local attention module

The global-local attention module is used to extract joint features that capture both global context and local details. It consists of two attention blocks: the global attention block (GAB) based on self-attention mechanism and the local attention block (LAB) based on channel spatial attention. The GAB learns global context information to provide a comprehensive understanding of the scene, enabling the localization of lesion regions and the suppression of irrelevant background information. The LAB refines local features to capture more detailed lesion information, which helps in distinguishing similar imaging features of complications in acute ischemic stroke (AIS) and addressing the issue of “different diseases, same images.”

Global context encompasses the implicit relationships between pixels and scene information in an image, providing a holistic perception of the scene. It has been widely used in various computer vision applications such as scene parsing and object detection (12–15). Intuitively, not all image content in MRI contributes to the final diagnosis, and irrelevant background information may even have a detrimental effect. However, by reasoning about the global scene, it becomes easier to detect and focus on the lesion region in the image. Existing methods primarily rely on convolutional neural networks (CNNs) for extracting MRI images features, which do not fully exploit and utilize the global context of the image. This is mainly due to the local nature of CNNs, which prevents them from learning the global context that aids in better lesion localization. Currently, effective modeling and integration of global context information in MRI images feature learning is an important research question that has not been fully investigated.

Inspired by natural language processing (NLP) networks, which extensively use transformers (14) to model global dependencies in language sequences, we designed a global attention block based on self-attention Transformer to model non-local interactions for learning global contextual information from local features. This global attention mechanism effectively detects lesion regions and efficiently disregards irrelevant background information. It is worth noting that, unlike segmentation-based methods, the GAB can locate lesion regions in an adaptive learning manner without any manual annotation. This enables more flexibility and robustness in handling various complex lesion shapes. The implementation process can be described as follows.

Taking into account that GAB required a sequence as input, similar to (15), the input feature map $F\in R^{C\times H\times W}$ was transformed into a sequence of flattened 2D patches $X_{p}\in R^{P^{2}C\times L}$, where (*P*, *P*) represented the resolution of each image patch, $L=HW/P^{2}$ was the number of image patches. Then, the image patches were mapped to a D-dimensional space through a learnable linear transformation, resulting in an output feature sequence $X\in R^{D\times L}$ which served as the input to GAB.

**Figure 3:**
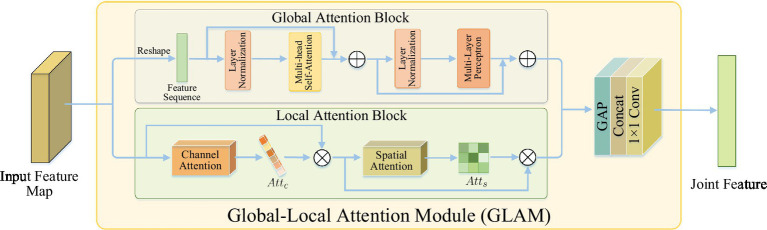
The structure of global-local attention module. Global attention block: learning global context information for detecting lesion regions; local attention block: refining local features to learn lesion details.

As shown in Figure 3, GAB consisted of two sub-layers: Multi-head self-attention (MHSA) and multi-layer perceptron (MLP). Each sub-layer was surrounded by residual connections, and layer normalization (LN) was applied before MHSA and MLP. Therefore, for an input feature sequence *X*, the output of GAB was given by


(1) $$F_{g}=X^{\prime }+\text{MLP}\left[\text{LN}\left(X^{\prime }\right)\right],$$

among,


(2) $$X^{\prime }=X+\text{MHSA}\left[\text{LN}\left(X\right)\right].$$

Although GAB can detect lesion regions through globally-guided attention, it is limited in learning lesion details. As the complications of AIS have similar imaging features, that is, there is a situation of “heterogeneous lesions and simultaneous shadows,” which requires learning more detailed lesion features to distinguish them. However, traditional CNN is difficult to accurately extract subtle visual features from images (16). The features extracted by CNN contain two types of information: channels and spatial regions, which have different contributions to learning lesion details (17, 18). In order to improve the characteristics of network contribution to high sensitivity, make it concentrate more high-end features, in order to enhance expressing ability for the pathological change information. In this study, a LAB based on channel spatial attention was designed to conduct adaptive feature refinement and refinement by learning the relationship between channel and feature spatial location.

As shown in Figure 3, LAB was constructed sequentially from channels and spatial attention to effectively aid the flow of information within the network by learning which information to emphasize or suppress. Based on this, the LAB can be adaptive to refine local characteristic lesions in order to obtain more detailed information, and thus improve the network’s ability to distinguish the similarity between classes of AIS complications.

Given a figure $F\in R^{C\times H\times W}$ as input, LAB sequentially output a channel attention map $Αtt_{c}\in R^{C\times 1\times 1}$ and a 2D spatial attention map $Αtt_{s}\in R^{1\times H\times W}$, which can be defined as


(3) $$\begin{array}{l} F^{\prime }={\text{Att}}_{c}\left(F\right)\circ F\\ F_{l}={\text{Att}}_{s}\left(F^{\prime }\right)\circ F^{\prime } \end{array}$$

In this context, ∘ represented the dot product, $F^{\prime }$ and $F_{l}$ correspond to the channel branch and LAB output, respectively.

#### 2.1.2 Multi-scale joint feature fusion module

Although many deep learning-based methods have achieved great success in stroke lesion detection, most of them rely on single-scale feature information and cannot fully utilize multi-scale feature representation, so it is difficult to deal with lesions of various scales and shapes, especially for small lesions (19, 20). This is because the following several pooling layer resolution is reduced, the characteristics of the small lesions may be lost. Therefore, this project designs a network with multi-scale feature information to realize the comprehensive extraction of MRI lesion features. It can combine the convolutional features of lesions of different scales and sizes together, retain low-level features of small lesions in the feature extraction process, and improve the diagnostic performance of early and mild patients.

In ALAM, a multi-scale feature extractor based on the pre-trained VGG16 (21) was first used to extract the feature representation of different layers. Then, at each scale, GLAM took the extracted feature map as input and learns joint features, implemented by two attention branches, capturing both global context and local refinement features. Finally, by integrating GLAM into the feature extractor, ALAM was designed to propagate joint features in a coarse-to-fine manner, fully extracted useful explicit feature representations at different scales, and exploit the complementary advantages of cross-scale implicit correlations. Therefore, the proposed network can effectively and robustly learn the imaging features of complex lesions by fusing multi-scale joint features, thereby significantly improving the detection performance of stroke lesions.

### 2.2 Based on the MLP patient metadata encoder

In clinical practice, radiologists will make medical diagnosis and evaluation of patients based on their imaging features and multi-dimensional clinical information database, including clinical data, past medical history, complications, etc. However, existing deep learning (DL) models only use a single imaging feature to predict the diagnosis of patients, which is not convincing and accurate. In view of this, in addition to imaging features, this project also established a multi-dimensional clinical information database, including clinical data, past medical history, complications, and self-care scores, which can provide patients with higher quality, more accurate and more personalized medical diagnosis AIS.

In order to make full use of patient metadata, this study selected 19 metadata features from the multi-dimensional clinical information database of patients, and designed a patient metadata encoder based on MLP to compile the patient metadata into the corresponding feature representation. Nineteen metadata features included “hospital,” “gender,” “age “, “disorder “, “consciousness,” “complications (hemiplegia) “, “pneumonia,” “aphasia,” “swallowing disorder,” “facial paralysis,” “dementia,” “cognitive impairment,” “depression,” “first ability score,” “hypertension,” “diabetes,” “atrial fibrillation,” “coronary atherosclerotic heart disease,” “hyperlipidemia,” “high homocysteinemia.” Specifically, there were two dense layers in this encoder, including 256 neurons. Each layer was followed by batch normalization and rectified linear unit (ReLU) activation layers. After the first dense layer attempts to generalize the metadata into the network, a further 25% was discarded using the dropout layer, thus avoiding possible overfitting of the data.

### 2.3 Cross-modal attention module

In order to strengthen the transfer between features and improve the performance of multimodal features, this study proposed a cross-modal attention module for multi-dimensional data information fusion such as clinical data, imaging features, treatment plans, prognosis and complications to achieve information complementarity and cross-validation. Multimodal fusion features not only retained the imaging features of MRI, but also made up for the clinical relevant information of patients, which provided more rich and comprehensive information for clinical diagnosis and treatment. The specific implementation process was as follows:

Firstly, the imaging features $F_{1}$ extracted by the adaptive lesion perception module and the metadata features $F_{2}$ obtained by the patient metadata encoder were combined into a multimodal joint feature ***F***:


(4) $$F=\text{Concat}\left(F_{1},F_{2}\right).$$

Then, it was considered that the operation of directly concatenating these two features might propagate a large amount of useless information and noise generated during the encoding process to the decoding layer. Therefore, this project proposed to model the relationship between features by cross-modal attention to generate attention masks, and used it to adaptively select important features, which could inhibit the spread of some harmful information to a certain extent. The attention mask ***A*** can be obtained from the following formula:


(5) Α=σ{FC{ReLU{ΒN[FC(F)]}}},

in which, σ and ReLU were sigmoid and rectified linear unit activation functions, fully connected layer (FC) and batch normalization (BN) were fully connected layer and batch normalization, respectively.

Finally, the learned attention mask A was multiplied with the original feature map F to generate a feature map with attention weights $F_{o}$:


(6) $$F_{o}=A\circ F,$$

in which, ∘ was the dot product. Based on this, the network can adaptively attention important characteristics, noise and suppress irrelevant information. These features were then fed into a classifier to predict the final outcome.

## 3 Experiment and result analysis

### 3.1 Data sets and experimental settings

#### 3.1.1 Dataset

This study collected 337 patients diagnosed with acute cerebral infarction and included MRI examinations from January 2019 to January 2023 in Panyu Central Hospital of Guangzhou. Please refer to Table 1 and Supplementary material for details of the data set.

**Table 1:** Distribution of cases (*n* = 337) and distribution of training test data (*n* = 3,106).

No.	Distribution pattern	Training set			Testing set		
		Eusemia	Poor prognosis	Total	Eusemia	Poor prognosis	Total
1.	Case distribution	142	95	237	60	40	100
2.	Training test data	1,136	977	2,113	497	396	893

#### 3.1.2 Experimental setup

This experiment was deployed in the PyTorch deep learning framework. The server used was equipped with two NVIDIA GeForce RTX 3090 GPUs with 24G memory. In the study, the input images are resize to 224 × 224 in both training and testing. During training, the cross-entropy loss function is applied to calculate the loss values between the predicted results and ground-truth labels. Moreover, the loss of the network is minimized by the Adam optimizer with a learning rate of 0.001, where the batch size is set to 32 and training is stopped after 200 epochs.

#### 3.1.3 Evaluation indicators

This article selected the accuracy, precision, sensitivity (i.e., recall), *F*1-score as a model of evaluation index, the calculation formula, respectively, as follows:


(7) $$\text{Accuracy}=\frac{\text{TP}+\text{TN}}{\text{TP}+\text{FP}+\text{TN}+\text{FN}}$$

(8) $$\text{Precision}=\frac{\text{TP}}{\text{TP}+\text{FP}}$$

(9) $$\text{Sensitivity}=\frac{\text{TP}}{\text{TP}+\text{FN}}$$

(10) $$\text{Specificity}=\frac{\text{TN}}{\text{TN}+\text{FP}}$$

(11) $$F1-\text{score}=2\times \frac{\text{Precision}\times \text{Sensitivity}}{\text{Precision}+\text{Sensitivity}}$$

Here, true positives (TP) and true negatives (TN) are the number of positive samples (that is, poor prognosis) and negative samples (that refers to good prognosis) that are correctly predicted, respectively. False positive (FP) is the number of negative samples misjudged as poor prognosis, and false negative (FN) is the number of poor prognosis samples misjudged as negative. In all experiments, the overall performance of the proposed and comparative methods was evaluated by calculating the mean and standard deviation of the cross-validation measures.

### 3.2 Experimental results

#### 3.2.1 Comparison of performance indicators of different models

To validate the performance of the proposed model, it was compared with advanced image classification frameworks, including Res2Net (20), ResNet50 (22), CSPNet (23), EfficientNet (24), HRNet (25) and VGG16-GLAM. VGG16-GLAM is regarded as the proposed MRI analysis model that only takes MRI as input. The performance metrics of different models were shown in Table 1. The proposed model achieved an accuracy of 91.17%, precision of 87.26%, recall of 95.21%, and F1-score of 91.06%, which significantly outperformed existing CNN frameworks. Compared to ResNet50 (22), the proposed model showed improvements of 6.04% in accuracy, 20.97% in recall, and 4.65% in *F*1-score. Although our method did not achieve optimal precision, it significantly improved the prediction of adverse prognosis in stroke patients compared to other methods. This has important clinical implications in the treatment of stroke patients, and it confirmed that the fusion of multidimensional data information, including clinical data, radiological features, treatment plans, prognosis, and complications, was beneficial for assessing the quality of life in patients with acute ischemic stroke.

**Figure 4:**
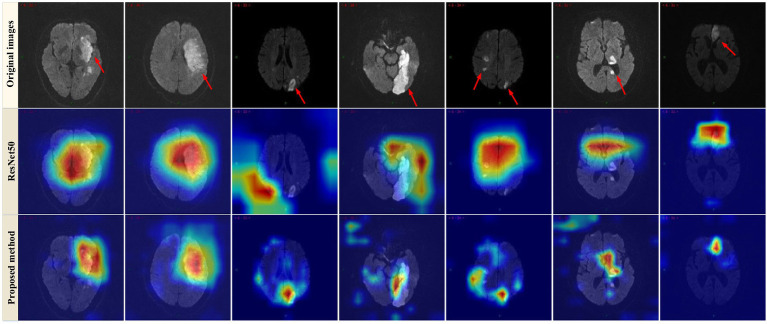
Examples of Grad-CAM thermal map of MRI. Original images (top row) and the Grad-CAM thermal map of the ResNet50 model (2nd row) and the proposed method (bottom row).

Furthermore, the Grad-CAM technique (26) was applied to visualize the proposed model. As observed in Figure 4, the Grad-CAM saliency maps of ResNet50 can roughly locate the lesion regions, but they may overlook or misjudge certain small lesions (e.g., the last two images in the last column). In contrast, the proposed model can accurately capture lesions of various complex shapes, even detecting and paying attention to minor abnormalities. This indicated that by learning joint features that encompass global context and local refinement, the model could adaptively detect relevant lesion regions to extract more discriminative feature representations, leading to better identification of patient case types, surpassing other state-of-the-art techniques. The experimental resulted in Figure 4 strongly support that the proposed model is intuitive and interpretable, confirming that the model’s decisions primarily depend on the lesion regions while disregarding irrelevant image content.

#### 3.2.2 Ablation experiments

In this subsection, we conducted a series of ablation experiments to validate the effectiveness of each key component of the prognostic assessment model for acute ischemic stroke based on multidimensional radiological data. Ablation studies for the proposed modules, including the multi-scale (MS) framework, GLAM, and the MLP. The No. 1 setting is the baseline network (i.e., VGG16) without these three modules, and then adding the to it as the No. 2 setting. GLAM is integrated into No. 2 to verify its effectiveness (No. 3). The No. 4 setting is the full version of the proposed network (see Table 2).

**Table 2:** Comparison of the performance indicators of the different models.

Method	Accuracy (%)	Precision (%)	Recall (%)	*F*1-score (%)
ResNet50	85.67 ± 1.12	91.87 ± 2.50	74.24 ± 1.76	82.12 ± 2.07
Res2Net	82.17 ± 2.77	82.24 ± 3.01	76.01 ± 3.28	79.00 ± 3.14
CSPNet	80.96 ± 2.35	82.10 ± 1.98	72.98 ± 2.77	77.27 ± 2.31
EfficientNet	82.08 ± 3.24	85.93 ± 2.75	70.96 ± 4.79	77.73 ± 3.49
HRNet	83.65 ± 2.79	84.72 ± 3.89	77.02 ± 2.52	80.68 ± 3.06
VGG16	83.87 ± 1.57	86.41 ± 2.89	75.50 ± 3.78	86.41 ± 3.28
VGG16-GLAM	90.82 ± 2.24	90.88 ± 2.86	88.13 ± 4.04	89.84 ± 3.34
Proposed method	91.71 ± 2.68	87.26 ± 1.39	95.21 ± 2.02	91.06 ± 1.58

##### 3.2.2.1 Effectiveness of the multiscale framework

**Figure 5:**
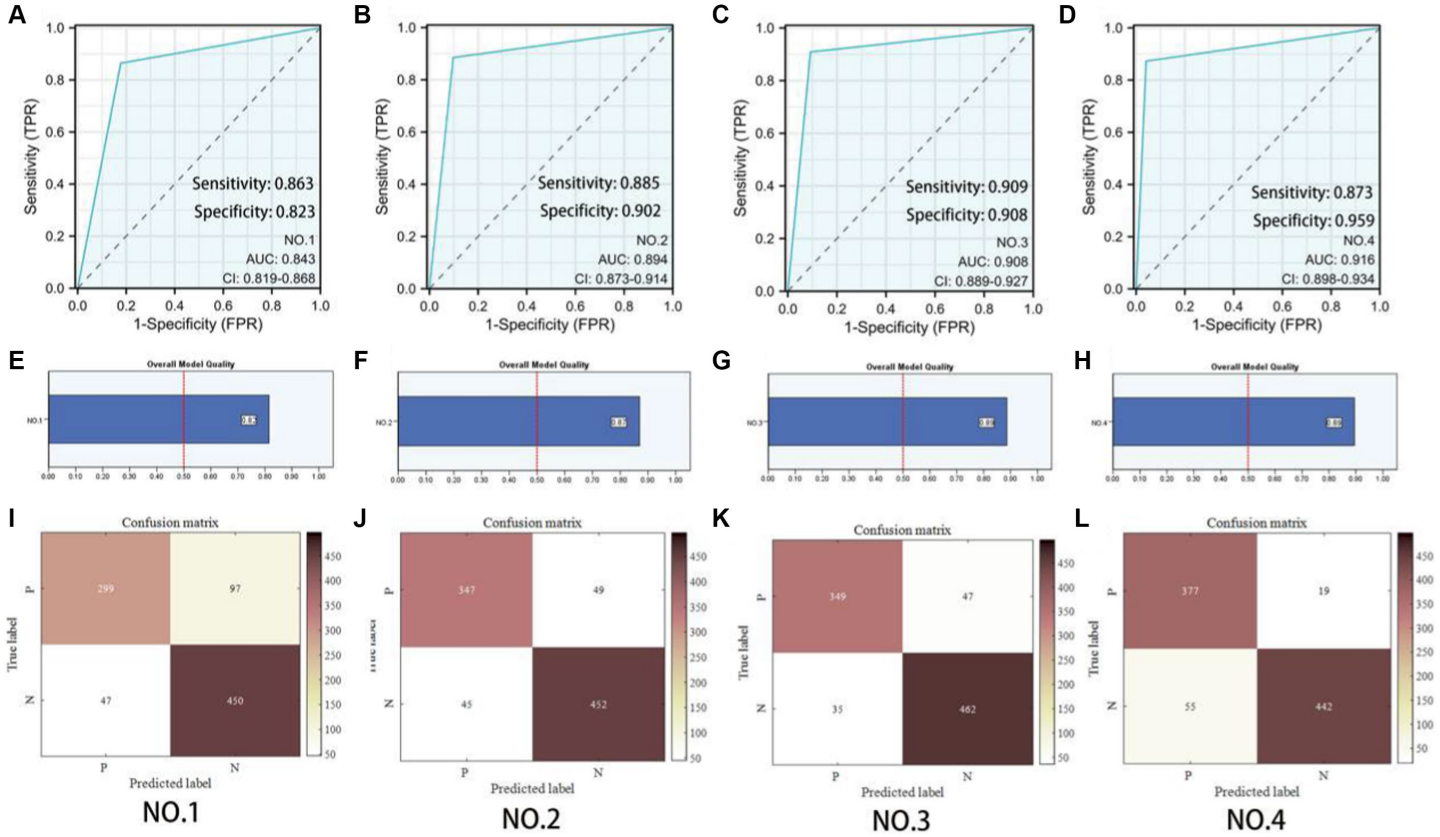
Predictive model evaluation. **(A–D)** Receiver Operating Characteristic curve. **(E–H)** Overall model quality. **(I–L)** Confusion matrixes of the ablation experiment. P: Poor prognosis; N: Eusemia.

To explore the contribution of the multiscale framework, we first used a single-scale network as the baseline model (No. 1) and compared it with other configurations. Table 3 showed that the proposed multiscale framework could learn better feature representations, resulting in performance improvements in all metrics compared to the single-scale framework. The No. 2 configuration using the multiscale framework achieved an accuracy improvement of 5.6%, sensitivity improvement of 12.12%, and precision improvement of 2.1%. The AUC of No. 1 and No. 2 was 0.843 and 0.894 respectively, and the model quality increased from 0.82 to 0.87. As seen in Figures 5A,B,E,F,I,J, the multiscale framework significantly enhanced the prediction of adverse prognosis in stroke patients. These results indicated that the multiscale framework was capable of capturing lesions of different scales and shapes, effectively leveraging the overall characteristics of brain MRI images.

**Table 3:** Effectiveness of component of the model.

No.	MS	GLAM	MLP	Accuracy	Precision	Recall	*F*1-score
1				83.87 ± 1.57	86.41 ± 2.89	75.50 ± 3.78	86.41 ± 3.28
2	√			89.47 ± 2.79	88.51 ± 1.02	87.62 ± 4.29	89.59 ± 1.65
3	√	√		90.82 ± 2.24	**90.88 ± 2.86**	88.13 ± 4.04	89.84 ± 3.34
4	√	√	√	**91.71 ± 2.68**	87.26 ± 1.39	**95.21 ± 2.02**	**91.06 ± 1.58**

Bold values indicate best performance.

##### 3.2.2.2 Effectiveness of GLAM

Compared to No. 2, the inclusion of the GLAM module in No. 3 effectively improved the recognition performance, with an accuracy improvement of 1.35%, sensitivity improvement of 0.51%, and precision improvement of 2.37%. The AUC of No. 2 and No. 3 was 0.894 and 0.908 respectively, and the model quality increased from 0.88 to 0.89. As shown in Figures 5B,C,F,G,J,K, the addition of the GLAM module enhanced the negative prediction level for stroke patients compared to Figure 5J. This indicated that the GAB in GLAM models the global contextual information, constructing global-guided attention to adaptively focus on affected regions while disregarding irrelevant information for better lesion identification. The LAB could adaptively refine local features to obtain detailed lesion information. The combination of these two modules effectively utilized joint characteristics to adaptively focus on lesion regions and learned more detailed imaging features, thereby achieving better diagnostic performance. This could serve as the foundation for clinical prognosis assessment in stroke patients.

##### 3.2.2.3 Effectiveness of the patient metadata encoder based on MLP

Compared to No. 3, the fusion of patient metadata in No. 4 effectively improved the recognition performance, with an accuracy improvement of 0.89%, sensitivity improvement of 7.08%, and *F*1-score improvement of 1.22%. The AUC of No. 3 and No. 4 was 0.908and 0.916 respectively, and the model quality was 0.89. As observed in the comparison between Figures 5C,D,G,H,K,L, while there was a slight decrease in the negative prediction level, there was a significant improvement in the positive prediction level for stroke patients. In terms of clinical significance, the improved accuracy in positive prediction was more meaningful than negative prediction. This indicated that the inclusion of multidimensional clinical information could provide higher quality and more accurate assessments for patients.

## 4 Discussion

In the past few decades, various machine learning techniques, including logistic regression (LR) (27), linear discriminant analysis (LDA) (28), support vector machines (SVM) (29), decision trees (DT) (30), random forests (RF) (31), and neural networks (32), have been applied. These methods heavily rely on feature engineering, such as shape, texture, and pixel intensity distribution (histogram) obtained from computer programs, which can be used to identify potential imaging-based biomarkers and serve as input for improved machine learning models (33). SVM has improved the identification of carotid atherosclerosis (CA) from magnetic resonance brain images and prevented ischemic stroke in 97.5% of patients (34). The RF algorithm combined with geodesic active contour (GAC) model can automatically segment cerebrospinal fluid (CSF) in CT images for early identification of brain edema, a major medical complication after ischemic stroke (35). The LR algorithm has aided in the analysis of CT angiography (CTA) lesions and the discrimination of mobile intraluminal thrombus and atherosclerotic plaques, assisting in the selection of stroke treatment plans, with a sensitivity of 87.5% (36). The use of artificial neural networks to predict inadequate perfusion and the presence of effective collateral circulation in CT perfusion scans can facilitate further treatment, achieving an accuracy of 85.8% in testing on CT perfusion images of 396 patients (37). Several studies have employed machine learning methods on various public datasets to address various stroke-related issues for better improvement of healthcare systems and stroke treatment plans (38). However, the traditional machine learning approaches require preprocessing of input features and manual extraction. Optimization of image features and susceptibility to interference from multimodal imaging need further exploration and improvement (39). Recently, deep learning, as an emerging artificial intelligence (AI) technique, has the ability to automatically capture hierarchical and complex features from raw input data (40–42). Deep neural networks, with multiple layers, simulate the perception of the human brain, transforming “low-level” to “high-level” representations, particularly in large-scale task solutions, in imaging classification, natural language processing, or bioinformatics (43, 44). In recent years, medical image processing has emerged as a hot research topic in deep learning, involving disease classification (45), lesion localization and segmentation, imaging reconstruction (46), and other tasks. Therefore, deep learning has been widely applied in stroke diagnosis and management, such as predicting clinical prognosis in AIS patients (47). Compared to traditional machine learning methods, deep CNN learning does not rely on handcrafted features. It automatically extracts and represents complex features when locating the core stroke lesions in CT or MRI (48). Deep learning not only saves time and effort but also captures pixel-level information of the lesions, contributing to improved diagnostic accuracy and prognosis (49).

Various artificial intelligence models have been widely applied in predicting the prognosis of ischemic stroke patients. Compared to predicting future stroke lesions on CT or MRI predicting patient prognosis is more challenging because commonly used prognostic scoring systems, such as the modified Rankin scale (mRS), are nonlinear and subjective, analyzing patients as a whole rather than on a voxel-by-voxel basis. This means there are fewer opportunities for artificial neural networks to learn from data, requiring larger training datasets to compensate for this limitation.

Previous studies mostly used non-imaging data as input and employed simple statistical models or machine learning models to predict prognosis (47, 50–53). However, CT or MRI can provide more information such as the size and location of infarctions. Tang et al. (54) utilized machine learning techniques combined with clinical data and the core-penumbra mismatch ratio from MRI and MRI perfusion to determine post-thrombolysis clinical outcomes. The short-term (7-day) result had an area under curve (AUC) of 0.863 [95% confidence interval (CI), 0.774–0.951], and the long-term (90 days) result had an AUC of 0.778 (95% CI, 0.668–0.888). Decision tree-based algorithms were able to predict the recovery outcomes (mRS >2 at 90 days) utilizing imaging and clinical data, with AUCs of 0.746 (extreme gradient boosting) and 0.748 (gradient boosting machine). Wang et al. (9) and Zhou et al. (8) used a multivariate logistic regression model to construct an imaging omics nomogram containing patient characteristics and imaging omics characteristics, and the AUC used to predict stroke outcome was greater than 0.80. Sun et al. (10) used clinical features and apparent diffusion coefficient maps to predict poor prognosis of acute stroke (mRS score >2) and the AUC was 0.80. These models were superior to models using non-imaging data, and the clinical data were continuous and related, which demonstrates the great potential of the combination. The performance of this algorithm further improved when National Institute of Health stroke scale (NIHSS) 24 h and reperfusion status were included (55). Machine learning techniques, including regularized logistic regression, linear support vector machine, and random forest, outperformed existing pre-treatment scoring methods in predicting favorable clinical outcomes (90 days mRS >2) for patients undergoing thrombectomy for large vessel occlusion (LVO) (50).

Osama et al. (56) developed a parallel multi-parameter feature embedding Siamese network (PMFE-SN) that can learn from a small number of samples and handle skewness in multi-parameter MRI data. The proposed multi-parameter embedding architecture in PMFE-SN is based on deep learning and avoids overfitting even with a small number of samples in the dataset. The authors successfully predicted the prognosis of acute ischemic stroke patients 3 months later using MRI perfusion images and clinical data from the 2017 Ischemic Stroke LEsion Segmentation (ISLES) challenge, demonstrating superior performance compared to other advanced techniques.

Hilbert et al. (57) compared a deep learning model constructed using residual neural networks with a machine learning model utilizing traditional radiological image markers. The results showed that automatic image analysis using deep learning methods outperformed previous radiological image markers in predicting the prognosis of ischemic stroke patients and had the potential to improve treatment selection.

The proposed multi-dimensional ADL scoring network framework for AIS patients has higher accuracy than other state-of-the-art network models. Ablation experiments also confirmed the effectiveness of each module in the framework. In addition, the visualization results using the Grad-CAM technique show that our method can accurately locate the lesion area while ignoring irrelevant background information, indicating that the final identification results determined by the model are reliable and interpretable. This will help to provide more rich and comprehensive information for providing clinical diagnosis and treatment.

The effect of allowing machines to autonomously learn to fit nonlinear equations based on massive data rather than artificial formulas is closer to the complex problem itself. The same deep learning network can be trained for different types of samples and produce different fitting models individually to enhance its general applicability. Therefore, the development of acute cerebral infarction prognostic risk prediction models based on imaging and multi-dimensional data based on PyTorch deep learning framework is of great significance for early evaluation and intervention, guiding treatment plans and judging prognosis, reducing disability rate and reducing social and economic burden.

A total of 337 patients were included in our study, including 237 in the training set and 100 in the testing set. A study by Quan et al. (58), such as using fluid attenuated inversion recovery (FLAIR) and apparent diffusion coefficient (ADC) images to extract the image of omics characteristics to predict the prognosis of patients with AIS, included 190 cases of acute ischemic stroke patients, divided into the training group (*n* = 110) and external validation group (*n* = 80). In the study by Tang et al. (59) to predict the prognosis of patients with acute ischemic stroke receiving thrombolytic therapy, 168 patients with acute ischemic stroke were included. Compared with these studies on predicting the prognosis of ischemic stroke, our study not only included a large sample size, but also had a richer content, not limited to a specific treatment (thrombolysis, mechanical thrombectomy, etc.) and a single subtype of ischemic stroke. The larger sample size in our study provided more statistical power and enhances the reliability of the findings. This broader scope allowed for a more comprehensive understanding of the factors influencing the prognosis of ischemic stroke patients.

However, it is important to acknowledge the limitations of our study. Firstly, the retrospective nature of the study design introduces inherent limitations. Retrospective studies rely on existing data, which may be subject to selection bias and confounding factors. Prospective studies would provide more robust evidence and minimize potential biases. Secondly, all the samples used in our study were obtained from a single center, which might limit the generalizability of the findings. The patient population and treatment protocols at a single center may not be representative of other centers or populations. Therefore, multicenter validation is necessary to confirm the external validity and generalizability of our results.

To address these limitations, future studies could employ prospective designs with larger and more diverse samples, including patients from multiple centers. In addition, the inclusion of additional clinical and imaging variables could further enhance the predictive accuracy of our models. By addressing these limitations and conducting more rigorous studies, we can strengthen the evidence base and improve the prediction of prognosis in patients with ischemic stroke.

## 5 Conclusion

In this study, a model consisted of ALAM, MLP-FE, CMAM were constructed for multi-dimensional data information fusion, such as clinical data, imaging features, treatment plans, prognosis and complications, so as to achieve complementary advantages. The fusion features not only retain the MRI images features, but also make up for the clinical relevant information of the patient, which provided higher quality, more accurate and more personalized medical diagnostic for prognosis of AIS.

## Data availability statememt

The original contributions presented in the study are included in the article/Supplementary material, further inquiries can be directed to the corresponding authors.

## Ethics statement

The Ethics Committee of Panyu District Central Hospital of Guangzhou reviewed and approved this study. The ethics committee waived the requirement of written informed consent for participation due to the retrospective nature of the study. The study was conducted in accordance with the local legislation and institutional requirements.

## Author contributions

JL: Data curation, Writing – original draft. JF: Methodology, Writing – original draft. ZL: Methodology, Writing – original draft. JW: Investigation, Writing – original draft. XL: Software, Writing – original draft. QW: Data curation, Writing – original draft. BH: Validation, Writing. HC: Project administration, Writing – original draft, Writing – review & editing. YY: Formal analysis, Writing – review & editing.
