# Supplementary material for: Research on prognostic risk assessment model for acute ischemic stroke based on imaging and multidimensional data
Source: Front Neurol. 2023 Dec 19;14:1294723. doi: 10.3389/fneur.2023.1294723 (PMC10773779; doi:10.3389/fneur.2023.1294723)
Supplement: Supplementary file 1 [file Data_Sheet_1.docx]

Clinical baseline data, including: basic information: name, gender, age; Risk factors of stroke: hypertension, diabetes, atrial fibrillation, coronary atherosclerotic heart disease, hyperlipidemia, hyperhomocysteinemia, use of anticoagulant and antiplatelet aggregation drugs, previous cerebral infarction, smoking, drinking; Imaging data: Brain MRI, MRA. TOAST classification of ischemic stroke (large atherosclerosis type, cardiogenic type, small vessel occlusion type, known etiology type, unknown cause type); Complications and others. Scoring: NIHSS score, mRS score, and self-care score. Provide hierarchical analysis basis and operational information for subsequent prediction models.

For patients undergoing clinical treatment, the Barthel Index Rating Scale is used to assess their ability to take care of themselves. The Barthel Index Rating Scale scoring criteria: The patient's BI score and grading were calculated within 24 hours of admission and before discharge. A total Barthel score of ≤ 40 points indicates severe dependence (level 1), a total score of 41-60 points indicates moderate dependence (level 2), a total score of 61-99 points indicates mild dependence (level 3), and a total score of 100 points indicates no need for dependence (level 4). Severe dependence means that the patient cannot take care of themselves completely, while moderate dependence means that the patient cannot take care of themselves in part of their life, Mild dependence means that a patient cannot take care of a very small part of their life on their own, and no dependence means that the patient is fully capable of taking care of themselves. The main outcome of this study is good functional outcomes. We will define mild dependence (level 3) and no dependence (level 4) as having a good prognosis, while severe dependence (level 1) and moderate dependence (level 2) as having a poor prognosis. For specific details of the dataset, please refer to Table 1 and supplementary materials. There are a total of 337 cases, each with 2-14 typical MRI images. As the number of MRI images inputted into the model is 1 each time, the same case can obtain input data ranging from 2-14. Based on this, we ultimately divided the samples into a training dataset (n=2113) and a testing dataset (n=893), with 1136 and 497 having good prognosis in the training and testing sets, respectively.

Table 1 Case distribution (n=337) and training test data distribution (n=3106) in the dataset

| No | Distribution | Training set | | | | Testing set | | |
| --- | --- | --- | --- | --- | --- | --- | --- | --- |
|  |  | Good Prognosis | Poor prognosis | Total | Good Prognosis | | Poor prognosis | Total |
| 1. | Case distribution | 142 | 95 | 237 | 60 | | 40 | 100 |
| 2. | Training-test dataset | 1136 | 977 | 2113 | 497 | | 396 | 893 |

Total number of cases in the supplementary material dataset (n=337)

| clinical features | Training set(n=237) | Testing set(n=100) |
| --- | --- | --- |
| Age | 69.1±10.7 | 67.3±9.8 |
| Female | 87 | 45 |
| Hospitalization frequency≤2 | 203 | 91 |
| Hypertension | 155 | 40 |
| Diabetes | 74 | 27 |
| Atrial Fibrillation | 17 | 11 |
| Coronary Atherosclerotic Heart Disease | 39 | 21 |
| Hyperlipidemia | 67 | 13 |
| Hyperhomocysteinemia | 15 | 6 |
| Secondary bleeding | 19 | 12 |
| Disturbance of consciousness | 11 | 3 |
| Complications (hemiplegia) | 171 | 69 |
| Pneumonia | 37 | 12 |
| Aphasia | 117 | 45 |
| Dysphagia | 16 | 7 |
| Cognitive impairment | 211 | 9 |
| Facial paralysis | 119 | 57 |
| Dementia | 12 | 1 |
| Depressed | 9 | 1 |
| Good prognosis | 142 | 60 |
| Poor prognosis | 95 | 40 |

[1] Liu F, Tsang RC, Zhou J, Zhou M, Zha F, Long J, Wang Y. Relationship of Barthel Index and its Short Form with the Modified Rankin Scale in acute stroke patients. J Stroke Cerebrovasc Dis. 2020 Sep;29(9):105033. doi: 10.1016/j.jstrokecerebrovasdis.2020.105033. Epub 2020 Jun 23. PMID: 32807445.
